# Nuciferine Attenuates Cancer Cachexia‐Induced Muscle Wasting in Mice via HSP90AA1

**DOI:** 10.1002/jcsm.13777

**Published:** 2025-04-01

**Authors:** Xueyan An, Lisha Ma, Yulan Bai, Chaoyue Chen, Ji Liu, Awaguli Dawuti, Kewu Zeng, Baoxue Yang, Bo Han, Abudumijiti Abulizi

**Affiliations:** ^1^ Key Laboratory of Xinjiang Phytomedicine Resource and Utilization, Ministry of Education, College of Pharmacy Shihezi University Shihezi China; ^2^ State Key Laboratory of Natural and Biomimetic Drugs Peking University Beijing China

**Keywords:** AKT–mTOR, cancer cachexia, muscle wasting, HSP90AA1, NF‐κB, nuciferine

## Abstract

**Background:**

Around 80% of patients with advanced cancer have cancer cachexia (CC), a serious complication for which there are currently no FDA‐approved treatments. Nuciferine (NF) is the main active ingredient of lotus leaf, which has anti‐inflammatory, anti‐tumour and other effects. The purpose of this work was to explore the target and mechanism of NF in preventing cancer cachexia‐induced muscle atrophy.

**Methods:**

The action of NF against CC‐induced muscle atrophy was determined by constructing an animal model with a series of behavioural tests, H&E staining and related markers. Network pharmacology and molecular docking were used to preliminarily determine the mechanism and targets of NF against CC‐induced muscle atrophy. The mechanisms of NF in treating CC‐induced muscle atrophy were verified by western blotting. Molecular dynamics simulation (MD), drug affinity responsive target stability (DARTS) and surface plasmon resonance (SPR) were used to validate the key target of NF.

**Results:**

After 13 days of NF treatment, the reduction of limb grip strength and hanging time in LLC model mice increased by 29.7% and 192.2% (*p* ≤ 0.01; *p* ≤ 0.001). Gastrocnemius and quadriceps muscles weight/initial body weight (0.98 ± 0.11 and 1.20 ± 0.17) and cross‐sectional area of muscle fibres (600–1600 μm^2^) of NF‐treated mice were significantly higher than those of the model group (0.84 ± 0.10, 0.94 ± 0.09, 400–800 μm^2^, respectively) (*p* ≤ 0.01; *p* ≤ 0.01; *p* ≤ 0.001). NF treatment also decreased the MyHC (myosin heavy chain) degradation and the protein levels of muscle‐specific E3 ubiquitin ligases Atrogin1 and MuRF1 in the model group (*p* ≤ 0.001; *p* ≤ 0.01; *p* ≤ 0.05). Network pharmacology revealed that NF majorly targeted AKT1, TNF and HSP90AA1 to regulate PI3K‐Akt and inflammatory pathways. Molecular docking predicted that NF bound best to HSP90AA1. Mechanism analysis demonstrated that NF regulated NF‐κB and AKT–mTOR pathways for alleviating muscle wasting in tumour bearing mice. The results of MD, DARTS and SPR further confirmed that HSP90AA1 was the direct target of NF.

**Conclusions:**

Overall, we first discovered that NF retards CC‐induced muscle atrophy by regulating AKT–mTOR and NF‐κB signalling pathways through directly binding HSP90AA1, suggesting that NF may be an effective treatment for cancer cachexia.

## 1 Introduction

As a multifactorial syndrome, cancer cachexia (CC) is defined as a persistent loss of skeletal muscle mass (with or without fat mass loss), which is primarily caused by aberrant metabolism and a variable combination of decreased food intake [1]. Studies have shown that about 80% of cancer patients have cachexia, which can directly cause as many as 40% of cancer‐related deaths [2]. The primary location of protein loss in CC is skeletal muscle [3]. The excessive protein catabolism of skeletal muscle leads to the reduction of quantity and strength of muscles [4]. Patients diagnosed with lung cancer, gastric cancer or colorectal cancer frequently experience cachexia, which manifests as substantial weight loss and a decline in overall quality of life [5]. Unfortunately, no effective treatment exists to restore muscle atrophy in cachectic cancer patients.

Despite the complexity of the pathological mechanism underlying CC, previous research has demonstrated that protein synthesis and degradation play a significant role in its development. Under normal circumstances, the process of synthesis and degradation in muscle tissue is balanced [6]. Activation of protein degradation pathways or mediators, such as nuclear factor‐kappa B (NF‐κB) and signal transducer and activator of transcription3 (STAT3), has been found to be associated with skeletal muscle mass loss [7]. NF‐κB activation leads to increased expression of E3 ubiquitin ligase, which will lead to the decomposition of MyHC and other components in sarcomere coarse myofilaments during atrophy [8]. In addition, the Akt–mTOR pathway is one of the key intracellular pathways controlling skeletal muscle mass, which is thought to primarily function by increasing protein synthesis via modifying translation initiation [9], indicating that the primary cause of muscle atrophy is an imbalance between the skeletal muscle's protein synthesis and degradation.

Nuciferine (NF), an active component of lotus leaf, has health‐promoting properties as a herbal medication [10], exhibits a wide range of pharmacological effects, including antitumour [11] and anti‐inflammatory properties [12]. Researchers found that NF inhibited inflammation induced by cerebral ischemia–reperfusion injury via NF‐κB signalling pathway [13]. The pharmaceutical effects and activities mentioned above may help relieve CC and enhance life quality [14]. However, the therapeutic effect and mechanism of NF on CC remain unclear.

Potential interactions between drug candidates and target receptors have been predicted using computer simulations [15]. Network pharmacology is a multidisciplinary field that explores the connections between drug components, targets and diseases by studying how pharmaceuticals interact with biological networks through pharmacological mechanisms [16]. Molecular docking, a computer simulation technology, can preliminarily predict the binding affinity and mode between small molecules and receptors [17]. Molecular dynamics simulation (MD) is a useful method for estimating how biomolecular complexes will bind and interact with each other [18]. Therefore, we employed computer simulations to further predict the targets and mechanism of NF against tumour cachexia‐induced skeletal muscle wasting.

In this study, the mechanism and targets of NF against CC‐induced skeletal muscle wasting were investigated using computer simulation in conjunction with experimental verification. First, we investigated the anticachexia effect of NF on LLC tumour‐bearing mice. Next, the hub targets and pathways of NF against CC‐induced muscle wasting were predicted by systems pharmacology with many databases. The results were confirmed through molecular docking and western blotting. Finally, we confirmed the target of NF against CC by MD, drug affinity response target stability (DARTS) and surface plasmon resonance (SPR).

## 2 Methods

### 2.1 Cell Culture

The high glucose DMEM complete media, which contains 10% FBS and 1% penicillin–streptomycin mixture, was used to culture lung cancer cells (LLC; LL/2; LLC1, Procell Life Science& Technology, Wuhan, China) in culture dishes. The cells were then maintained at 37°C in an incubator with 5% CO_2_. Trypsin‐digested LLC cells grown to 80%–85% fusion were passaged cultured and expanded to sufficient numbers. Following digestion, centrifugation and cell passage, the cells were collected, rinsed with PBS and resuspended at a density of 5 × 10^6^/mL.

Mouse C2C12 myoblasts (Sevier Biotechnology, Wuhan, China) were cultured in high glucose DMEM containing 10% FBS at 37°C under 5% CO_2_. When the cells reached 80% confluence, the medium was changed to differentiation medium (DMEM with 2% horse serum), and the cells were fused into multinucleated myotubes within 3 days of differentiation and were used for subsequent experiments.

### 2.2 Animals

Male C57BL/6 mice at 6–8 weeks old were purchased from Nanjing Junke Bioengineering Corporation, Ltd (Nan Jing City, Jiang Su Province, China). The mice were housed in a controlled setting with standard temperature (22 ± 1°C) and were freely provided with standard rodent water and food. The laboratory animal ethics committee at ShiHeZi University has approved this work (Approval Number: A2022‐090‐01, 2022).

### 2.3 CC Model and Drug Therapy

Briefly, the mice were randomly assigned to three groups: the normal control group (CON), the group of mice with LLC tumour (LLC) and the group of LLC tumour‐bearing mice that were intraperitoneally treated with 30 mg/kg/day of NF (LLC + NF). NF (DSTDH005101; greater than 98%, HPLC) was purchased from Lometian Medicine/Durst Bio (Chengdu, China). Following the dissolution of NF in 1 mol/L hydrochloric acid, sodium hydroxide was utilized to adjust the pH to 7.5 [19]. At the beginning, in the normal control group, mice received an injection of 200‐μL normal saline under the right subcutaneous axilla, and each mouse in the other two groups was injected with LLC cells (1 × 10^6^ cells/200 μL) under the subcutaneous axilla to observe the growth of tumours. When obvious tumours could be palpated, the LLC + NF group was injected intraperitoneally with NF (30 mg/kg/day) daily. Mice in the control and LLC groups received the same volume of vehicle (1 mol/L hydrochloric acid adjusted to pH 7.5 with sodium hydroxide). Weight, food consumption and tumour volume were assessed every 3 days. Tumour volume was measured by using a digital calliper and calculated tumour volume (*V* = x*x*y*0.52). During the intervention days, behavioural assessments were conducted on the mice before sacrifice. The mice were euthanized after anaesthesia with 1% pentobarbital. Then, the weight of the tumour, skeletal muscle tissue and epididymal fat were measured and recorded. For additional research, a part of the muscle tissue was rapidly frozen in liquid nitrogen.

### 2.4 Behaviour Tests

#### 2.4.1 Grip Strength Test (GST)

We used a commercial digital grip strength metre with an attached metal grid (ZS‐ZL, Beijing, China) to measure the muscle strength of the mice. On Days 7 and 13, to enable the four paws of the mice to grasp the wire grid connected to a dynamometer, they were hoisted and held by their tails. The tail subsequently drew the mice in a gradual reverse direction until they loosened their grips. Then, the peak force of the four limbs was recorded. The outcomes of three experiments conducted on each mouse were incorporated into a statistical analysis.

#### 2.4.2 Wire Grip Test (WGT)

On Day 12, each mouse permitted to grip a metal wire, and their clutch was timed until they slipped off. The average of at least three retakes was used to compute test results. Frailty scores were recorded as follows: 0 points: falling off the wire in less than 10 s; 1 point: lifting one of the hindlimbs; 2 points: attempting to climb the wire; 3 points: grasping the wire with both front and rear claws; 4 points: wrapping the legs and tail around the wire; and 5 points: attempting to get to the end of the wire.

### 2.5 Histology Examination

Tibialis anterior muscle was dehydrated and embedded in paraffin after being fixed with 4% paraformaldehyde. Next, the paraffin blocks were sliced into SM sections that were 5 μm thick. The muscle sections were then dewaxed, rehydrated and stained with haematoxylin and eosin (H&E). Finally, the slides were examined using a light microscope and micrographs were captured.

### 2.6 NF and CC Target Collection

NF's (Compound CID: 10146) chemical structure and calculated characteristics were taken from the PubChem database (https://pubchem.ncbi.nlm.nih.gov/). In this study, we collected the action targets of NF from TCMSP database (https://old.tcmsp‐e.com/tcmsp.ph), Swiss target prediction database (http://swisstargetprediction.ch/) and the UniProt (https://www.uniprot.org/) database transformed the target name into the gene symbol. Furthermore, gene targets of CC‐induced muscle wasting or CC‐induced muscle atrophy were collected from GeneCards database (https://www.genecards.org/) and Omim (https://www.omim.org/) and removed duplicated disease targets. Ultimately, Venn diagrams were employed to examine the gene targets of NF against CC‐induced muscle atrophy.

### 2.7 Constructing Protein–Protein Interaction (PPI) Network

The data were obtained and saved in Tsv format through the utilization of the String database. The database contained information on *Homo sapiens* species and proteins that correlated to genes with a high degree of confidence, as determined by a minimum interaction score of > 0.7. To visualize the PPI network diagrams, we imported the data into Cytoscape3.9.1 software. We used software to construct a PPI network diagram, and the top core proteins were selected for molecular docking.

### 2.8 GO and KEGG Enrichment Analysis

The compound‐disease key targets were uploaded to the DAVID database to analyse their main biological processes and pathways through enrichment analysis, and the data were downloaded and visualized with the microbiology platform (http://www.bioinformatics.com.cn/).

### 2.9 Molecular Docking

The 3D structure of the NF was acquired from the PubChem database (https://pubchem.ncbi.nlm.nih.gov) and saved in pdbqt format using AutoDockTools1.5.7 software. Protein structures were obtained from the PDB (http://www.rcsb.org) database and were searched by ‘Experimental’ and ‘ *Homo sapiens* ’. The protein 3D structures were screened using ‘Refinement Resolution 1.0‐2.0’ as the screening condition and single chain was preferred. The obtained proteins were processed with DiscoveryStudio2019 software to set the binding site size, and amino acid residues were removed by hydrogenation using AutoDockTools1.5.7 software and saved in pdbqt format. Finally, AKT1 (grid size: *x* = 22.78366, *y* = −15.7742, *z* = −15.9523, box size: 60), TNF‐α (grid size: *x* = 42.3537, *y* = 45.1851, *z* = 4.77167, box size: 54), EGFR (grid size: *x* = 4.46082, *y* = 8.20951, *z* = 14.9334, box size: 64), HSP90AA1 (grid size: *x* = −32.1761, *y* = −11.4867, *z* = −27.4536, box size: 42), MMP9 (grid size: *x* = 1.9282, *y* = 50.1767, *z* = 14.4835, box size: 42), mTOR (grid size: *x* = 7.84215, *y* = −22.0282, *z* = −34.7103, box size: 72), ICAM1 (grid size: *x* = −29.8672, *y* = −53.753, *z* = −7.24762, box size: 56.6), PPARG (grid size: *x* = 13.7456, *y* = 5.4313, *z* = 2.55286, box size: 37.2), PTGS2 (grid size: *x* = 19.1032, *y* = 43.2163, *z* = 44.1997, box size: 77.2), MMP2 (grid size: *x* = 3.85679, *y* = −16.3274, *z* = −8.07063, box size: 50), BCL2 (grid size: *x* = −16.1286, *y* = 10.9992, *z* = −12.8488, box size: 38) and ERBB2 (grid size: *x* = 18.5514, *y* = −4.73196, *z* = −1.71084, box size: 110) were docked with NF using vina, and the final docking scores were obtained. Finally, the docking was visualized using DiscoveryStudio2019 and PyMoL 2.5 software.

### 2.10 Western Blotting

According to the manufacturer's instructions, RIPA buffer (R0010, Solarbio, Beijing, China) with protease and phosphatase inhibitors (P1265‐2, P1260‐5, APPLYGEN, Beijing, China) was used to lysate skeletal muscle tissue. BCA protein assay kit (PC0020, Solarbio, Beijing, China) was used to quantify protein samples. The SDS‐PAGE gel was used to separate the protein samples, which were then transferred to a polyvinylidene fluoride membrane (Roche, Switzerland). Following the blocking process, the membranes were subjected to overnight incubation at 4°C with homologous primary antibodies aimed at GAPDH (1:40 000, Proteintech, 60004‐1‐Ig, Wuhan, China), AKT(1:1000, Cell Signaling Technology, 92772S, MA, United States), p‐AKT (Ser473,1:1000, ABclonal, AP0140,Wuhan, China), p‐mTOR (Ser2448,1:1000, Cell Signaling Technology, 5536T, MA, United States), mTOR (1:1000, Cell Signaling Technology, 2983T, MA, United States), HSP90AA1 (1:250, ABclonal, A23880, Wuhan, China), TNF‐α (1:1000, ABclonal, A0277, Wuhan, China), p‐IKKβ (Ser177/181, 1:1000, Cell Signaling Technology, 2694T, MA, United States), IKKβ (1:1000, Cell Signaling Technology, 2678T, MA, United States), IL‐6 (1:1000, ABclonal, A0286, Wuhan, China), NF‐κB (1:5000, Abmart, T55034S, Shanghai, China), p‐NF‐κB (Ser536,1:1000, Cell Signaling Technology, 3033T, MA, United States), MyHC (1 μg/mL, R&D Systems, CAEI0822101, MN, United States), Atrogin1 (1:1000, Abways, CY8766, Shanghai, China) and MuRF1 (1:1000, Proteintech, 55 456‐1‐AP, Wuhan, China), and then, an ECL plus kit was used to produce the blots (Proteintech, Wuhan, China). A chemiluminescence detection system (Syngene, Gene Gnome XRQ, Cambridge, Cambridgeshire, United Kingdom) was utilized to visualize the protein bands, and ImageJ software (NIH, MD, United States) was utilized for analysis.

### 2.11 MDs of NF‐Target Complex

The NF‐target complex with the highest score in molecular docking results was selected for MD using the GROMACS 2021.5 software package. The OPLS‐AA/L force field was employed for the protein, and the LigParGen service (http://zarbi.chem.yale.edu/ligpargen/) was performed to prepare the tiny molecular ligand. Then, the 3‐point simple point charge (SPC 216) water box model was used to place the simulation system. MD simulation was performed in a cubic box with a buffer distance of 10 Å on the outer surface of the protein. Sodium or chloride ions were randomly introduced in sufficient quantities to maintain the system's electrical neutrality. Subsequently, 1000 ps NVT and NPT balances were carried out. The protein‐ligand complex was produced using MD simulation for up to 50 ns. As a result, the trajectory was used to derive several geometric parameters of the system for further investigation, including hydrogen bond number, root mean square deviation (RMSD) and root mean square fluctuation (RMSF). Molecular mechanics Poisson–Boltzmann surface area (MM‐PBSA) method implemented in the GROMACS compatible tool ‘gmx_mmpbsa’ was utilized to calculate the binding free energies (ΔGbind).

### 2.12 SPR

In order to conduct SPR tests, a Biacore T200 instrument (GE Healthcare, Sweden) was employed. Flow Cell 1 was the reference channel, whereas Flow Cell 2 was the detecting channel. The surfaces of CM5 chips were coated with carboxymethylated dextran and then activated using a 1:1 mixture of N‐hydroxysuccinimide (NHS) and 1‐ethyl‐3‐[3‐dimethylaminopropyl] carbodiimide HCl (EDC). After being diluted in 10‐mM sodium acetate (pH 4.5, 20 μg/mL), the recombinant HSP90AA1 protein was drawn to the dextran matrix and covalently linked to it at a contact period of 900 s and a flow rate of 10 μL/min. The loosely attached ligands were further washed away and rendered inactive using ethanolamine. NF was diluted in a gradient from 256 to 16 μM after being dissolved in PBS containing 5% DMSO. At a flow rate of 30 μL/min, samples were introduced via the HSP90AA1 immobilized chip surface. The duration of contact and dissociation were, correspondingly, 60 and 120 s. The Biacore T200 evaluation software was applied to determine *K*_*D*_, and a steady‐state affinity model (1:1) was employed to suit the affinity curves.

### 2.13 DARTS

After collecting the C2C12 myotubes, lysis was performed using NP‐40 lysis buffer (Catalogue N8032, Solarbio, Beijing, China). Following this, TNC buffer was added to the lysis buffer. After aliquoting the lysate into 1.5‐mL containers, it was treated with NF or DMSO at room temperature for 1 h at varying concentrations. Following the incubation period, the lysates underwent digestion for 20 min using pronase E (catalogue P8360, Solarbio, Beijing, China) solution. Following the immediate addition of protein loading buffer, the lysates were heated to halt proteolysis. Western blotting was employed to conduct further examination.

### 2.14 Statistical Analysis

The values were provided as mean ± SD. Statistical significance was determined between groups using one‐ or two‐way ANOVA in Prism 8.4 software, with *p* < 0.05 being significant.

## 3 Results

### 3.1 NF Improved Muscle Strength of Tumour‐Bearing Mice

**FIGURE 1:**
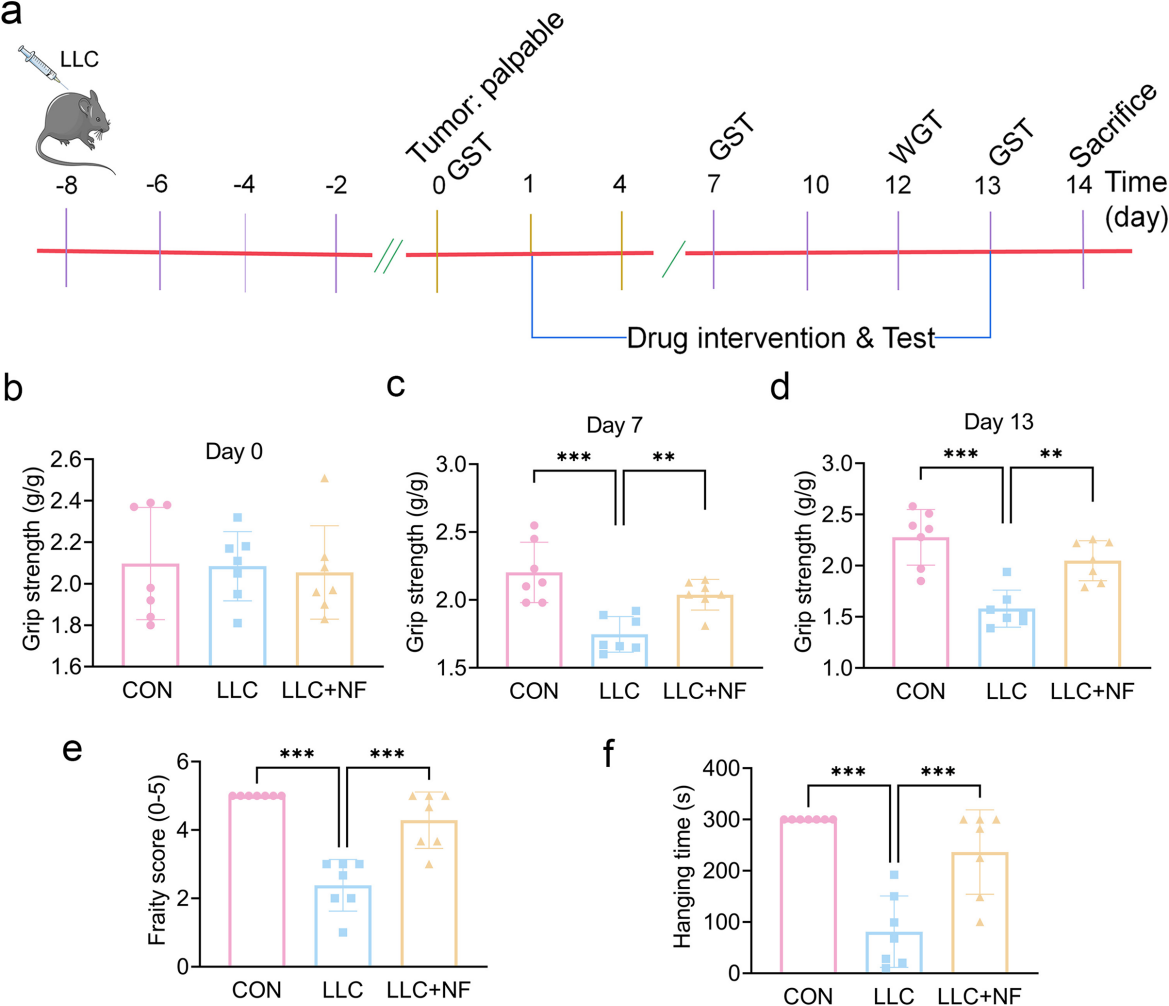
NF improved the muscle strength of LLC tumour‐bearing mice. (a) Study design. Tumours were inoculated on Day −8, tumours grew on Day 0, NF treatment was given on Days 1–13 and mice were sacrificed on Day 14. (b–d) Grip strength test (GST). Grip strength on Days 0, 7 and 14. (e, f) Wire grip test (WGT). Frailty score and hanging time on WGT. The data are shown as the mean ± SD. *n* = 7 mice/group. **p ≤ 0.01, ***p ≤ 0.001.

The continuous decline of muscle strength is a significant characteristic of tumour cachexia [20]. Tumour‐bearing mice were given NF (30 mg/kg/d, ip) for 13 consecutive days in order to examine the muscle strength and muscle atrophy (Figure 1a). The results showed that NF had no significant improvement in body weight with tumour‐bearing, food intake, tumour volume and tumour weight in tumour‐bearing mice (Figure S1a–d). Behavioural tests were performed to evaluate the changes in muscle function in LLC mice. The limb GST was conducted on the 0th, 7th and 13th days after administration. According to the experimental results, muscle strength was the same on Day 0 (Figure 1b). However, muscle grip strength was considerably decreased in the LLC group on Days 7 (Figure 1c) and 13 (Figure 1d) compared to the control group, and NF improved the muscle grip strength in the LLC group. On Day 12, there was a notable reduction in fore‐limb hanging time (Figure 1e) and frailty score (Figure 1f) in the LLC group in comparison to the control group. However, the LLC group treated with NF significantly alleviated those changes. These results suggest that NF can successfully boost muscle strength in mice bearing tumour.

### 3.2 NF Ameliorated the Loss of tumour free Body Weight, Muscle Mass and Skeletal Muscle Wasting of Tumour‐Bearing Mice

**FIGURE 2:**
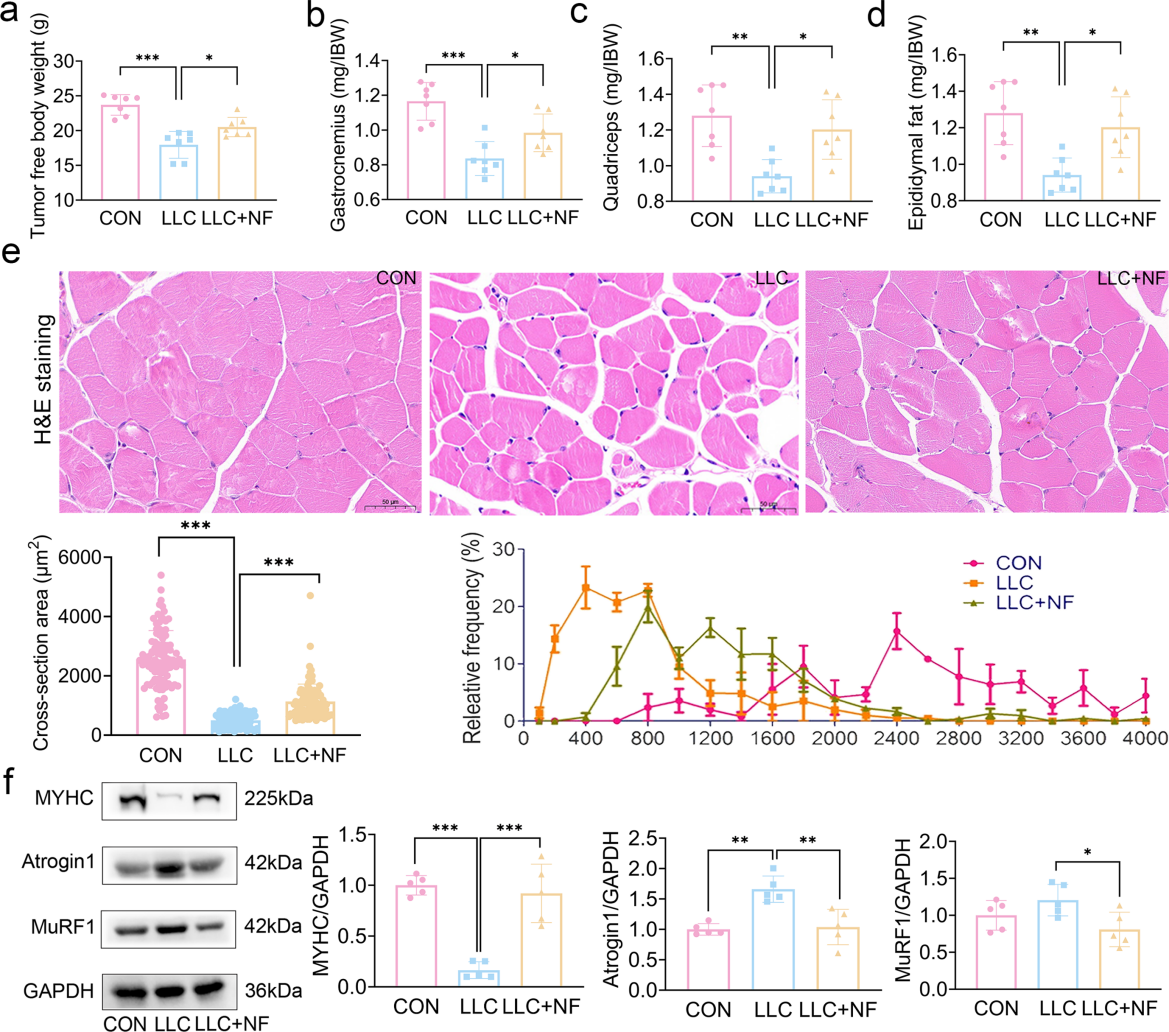
NF ameliorated the loss of tumour free body weight, muscle mass and skeletal muscle atrophy in mice bearing lung tumour: (a) The tumour free body weight. (b, c) Gastrocnemius muscle weight, quadriceps muscle weight to initial body weight (IBW). (d) Epididymal fat mass to initial body weight (IBW). (e) H&E‐stained sections of mouse muscle (scale bar = 50 μm), skeletal muscle myofiber cross‐sectional area and relative frequency. (f) Representative Western blots showing the expression of MYHC, Atrogin1, MuRF1 in the gastrocnemius muscle and relative protein levels in the experiments of MYHC, Atrogin1 and MuRF1. The data are shown as the mean ± SD. *n* = 7, 3 or 5 mice/group. *p ≤ 0.05, **p ≤ 0.01, ***p ≤ 0.001.

Loss of body weight, skeletal muscle and adipose tissue is frequently associated with CC [3]. Consequently, the mass of the tumour‐free body weight, skeletal muscle and epididymal fat was measured. Consistent with previous reports, we observed that the LLC group had significantly less tumour free body weight (Figure 2a), gastrocnemius mass (Figure 2b), quadriceps mass (Figure 2c) and epididymal fat mass (Figure 2d) than the control group. After NF treatment, the loss of tumour free body weight, quadriceps muscle, gastrocnemius muscle and epididymal fat mass in LLC tumour bearing mice were considerably improved, suggesting that NF could alleviate the tumour‐bearing mice's cachexia symptoms. H&E staining results showed that skeletal muscle cross‐sectional area (CSA) in the LLC group was significantly lower than in the control group (Figure 2e), whereas NF therapy significantly enhanced CAS in mice bearing‐LLC tumour. In the control group, myofibers were predominantly scattered between 1600–2800 μm^2^, but in the LLC group, they were more evenly distributed between 400–800 μm^2^. After NF treatment, the majority of myofibers in LLC tumour‐bearing mice measured between 600 and 1600 μm^2^ (Figure 2e). In addition, NF treatment significantly decreased MyHC degradation and activation of Atrogin1 and MuRF1 (muscle‐specific E3 ubiquitin ligases participated in protein degradation) when compared to the model group (Figure 2f). These findings showed that NF might successfully prevent skeletal muscle atrophy of LLC tumour‐bearing mice.

### 3.3 Network Pharmacology Analysis

#### 3.3.1 Target Prediction of NF and CC

**FIGURE 3:**
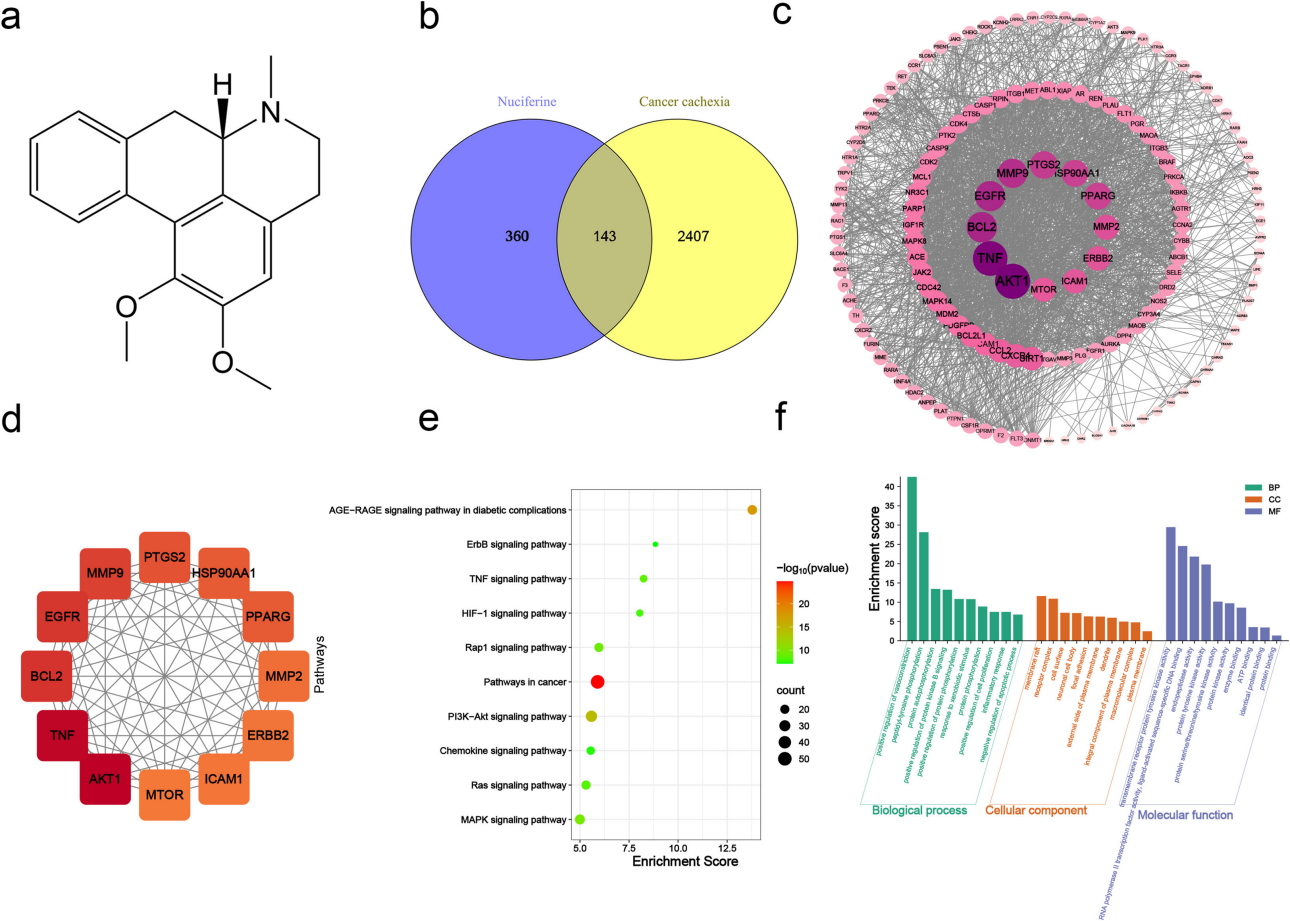
Network pharmacology analysis of NF on cancer cachexia. (a) The 2D structure of NF. (b) Common targets for the action targets of NF and CC. (c) PPI network diagram in which the size of the circle and the shade of the colour are arranged according to the degree. (d) The core target and the colour of the shade of the degree are arranged from largest to smallest. (e) KEGG enrichment analysis. (f) NF against cancer‐associated muscular dystrophy gene ontology enrichment analysis of potential targets. The top biological processes (BP), cellular components (CC) and molecular functions (MF) are shown as green, orange and purple bands, respectively.

We got the chemical structure of NF from the PubChem database (Figure 3a). Three‐hundred and sixty targets of NF were collected from TCMSP, Swiss target prediction and other databases; 2407 disease targets were collected from TTD, GeneGards and other databases; and 143 active ingredient‐disease cotargets were obtained by Venn diagram mapping (Figure 3b).

#### 3.3.2 PPI Network Analysis

Based on STRING results from 143 overlapping targets with high confidence (> 0.7), a ‘protein–protein network’ was constructed to further anticipate the correlations between targets of NF and CC. It was discovered that 146 nodes and 2030 edges were built in the PPI network, which represented targets and protein interactions, respectively (Figure 3c). The degree value was used for identifying the primary targets. Results showed that AKT1, TNF, BCL2, EGFR, MMP9, PTGS2, HSP90AA1, PPARG, MMP2, ERBB2, ICAM1 and mTOR were the top key targets (Figure 3d and Table S1).

#### 3.3.3 KEGG Pathway Enrichment Analysis and Target‐Pathway Network Analysis

As shown in Figure 3e, we further screened and summarized the KEGG pathways in depth, where the *Y* axis showed the specifics of each KEGG pathway whereas the *X* axis showed enrichment score of each pathway. Based on the *p* value, we screened the top 10 pathways in this study, including the pathways in cancer, PI3K‐Akt and MAPK. These pathways suggest that NF may mitigate CC‐induced wasting of muscles by regulating multiple genes and signalling pathways.

#### 3.3.4 Gene Ontology (GO) Enrichment Analysis

Next, we used the DAVID database (https://david‐d.ncifcrf.gov/) to perform GO enrichment analysis in order to forecast the biological processes, cellular components and molecular activities of the key targets. A number of biological processes, including signal transduction, positive regulation of cell proliferation, protein phosphorylation and inflammatory response, were implicated. The cellular component includes the plasma membrane and an integral component of the plasma membrane. It takes part in various molecular processes such as protein binding, ATP binding and identical protein binding (Figure 3f), suggesting that inflammatory response and positive regulation of cell proliferation are the main biological processes for the role of NF against CC.

### 3.4 Molecular Docking

**FIGURE 4:**
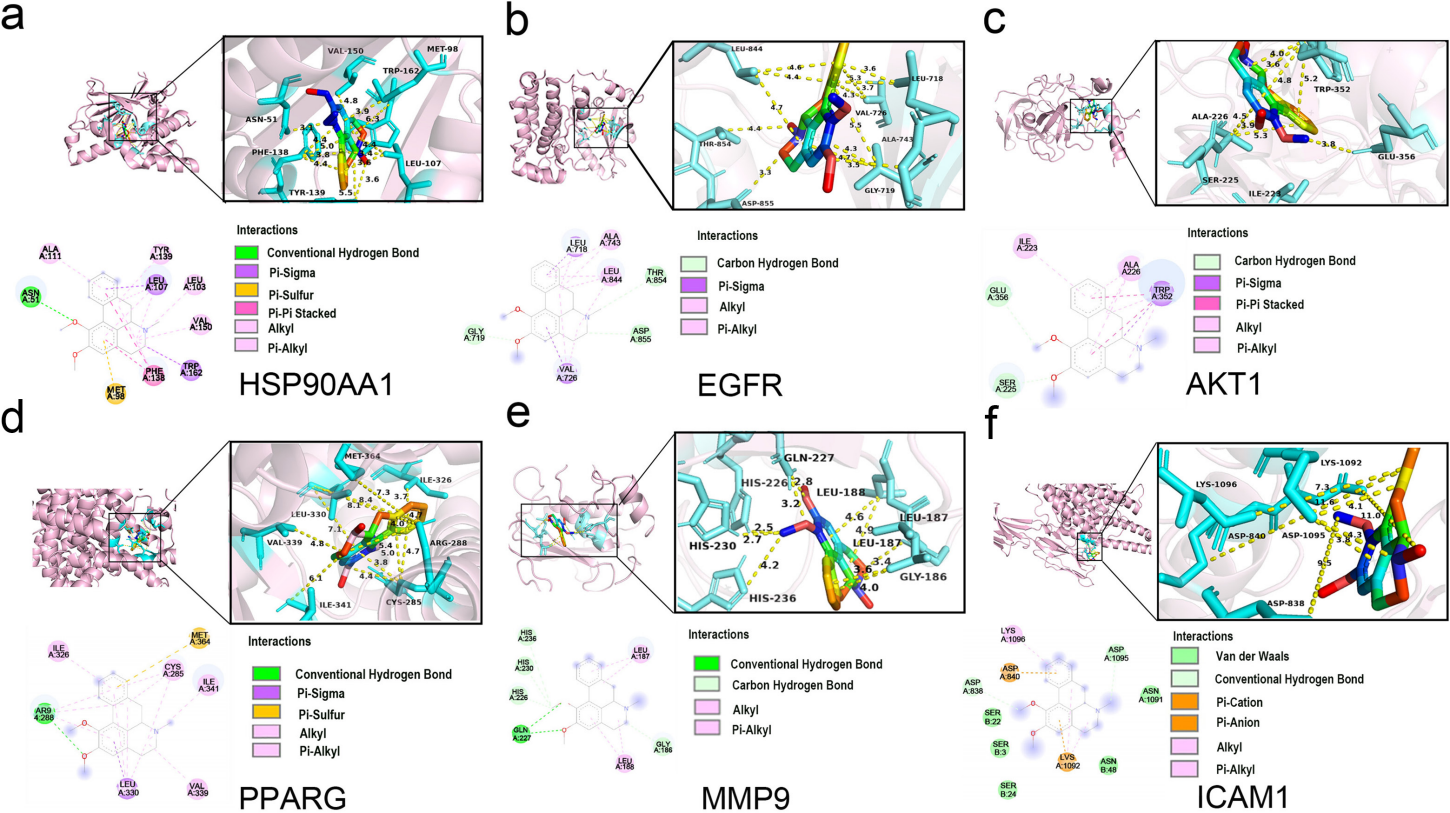
Visualization was performed based on molecular docking results. NF docked with (a) HSP90AA1, (b) EGFR, (c) AKT1, (d) PPARG, (e) MMP9 and (f) ICAM1.

We performed molecular docking to determine the binding between screened main targets and NF to validate the consequences of network pharmacology. On the basis of KEGG pathway enrichment analysis and PPI network degrees, we finally selected the top target genes for molecular docking. According to the molecular docking research, NF's binding energies with the aforementioned key targets ranged from −9.5 to −6.3 kcal/mol (Table S2), indicating that NF had a good binding with those targets. The results showed that NF had the strongest binding affinity to HSP90AA1, followed by EGFR, AKT1, PPARG, MMP9, ICAM1, PTGS2, MMP2, BCL2, TNF‐α, mTOR and ERBB2. The specific docking sites of NF and target proteins are shown in Figure 4 and Figure S2.

### 3.5 NF Inhibited Inflammation Response of Muscle via HSP90AA1/NF‐κB Pathway in Lung Tumour‐Bearing Mice

**FIGURE 5:**
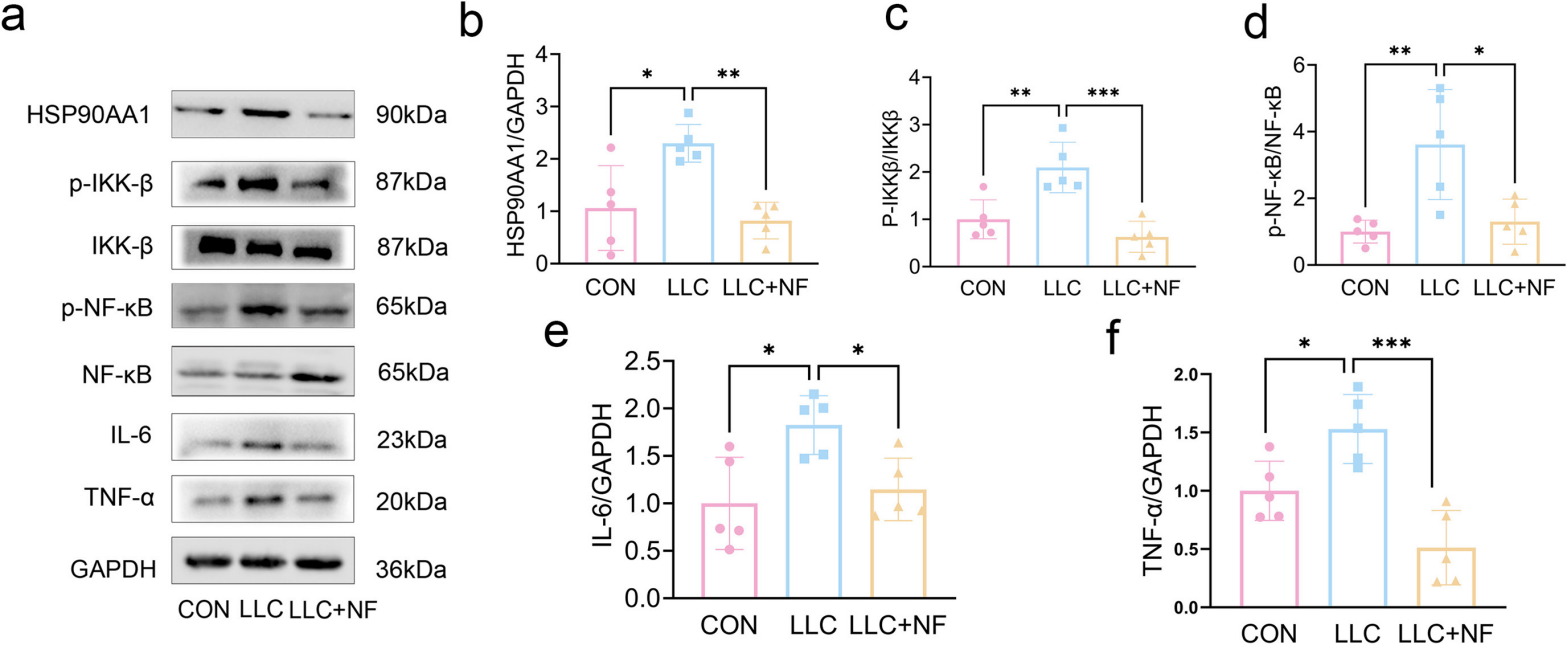
NF inhibited inflammation response of muscle via HSP90AA1/NF‐κB pathway in LLC tumour‐bearing mice. (a) Representative Western blots showing the expression of HSP90AA1, p‐IKKβ, p‐NF‐κB, IL‐6 and TNF‐α in the gastrocnemius muscle. (b–f) Relative protein levels in the experiments shown in A. The data were shown as the mean ± SD, *n* = 5. *p ≤ 0.05, **p ≤ 0.01, ***p ≤ 0.001.

According to network pharmacology and molecular docking, HSP90AA1 and TNF‐α may be implicated in the action of NF against the wasting of muscles in tumour‐bearing mice. The GO enrichment analysis suggested that one of the primary biological mechanisms through which NF protected against CC was the inflammatory response. Therefore, we used Western blot to further evaluate inflammation‐related pathway. Our findings demonstrated that, in comparison to the control group, the expression of HSP90AA1, p‐IKKβ/IKKβ, p‐NF‐κB/NF‐κB, TNF‐α and IL‐6 in the model group increased. Luckily, NF successfully reduced expression of the above proteins in tumour‐bearing mice (Figure 5a–f). The above findings indicated that activation of inflammation, indeed, plays a significant role in skeletal muscle wasting in LLC tumour‐bearing mice. Luckily, NF may mitigate CC‐induced muscle wasting by inhibiting the inflammation through inhibiting the HSP90AA1*/*NF‐κB signalling pathway.

### 3.6 NF Attenuated CC‐Induced Muscle Loss by AKT–mTOR Pathway

**FIGURE 6:**
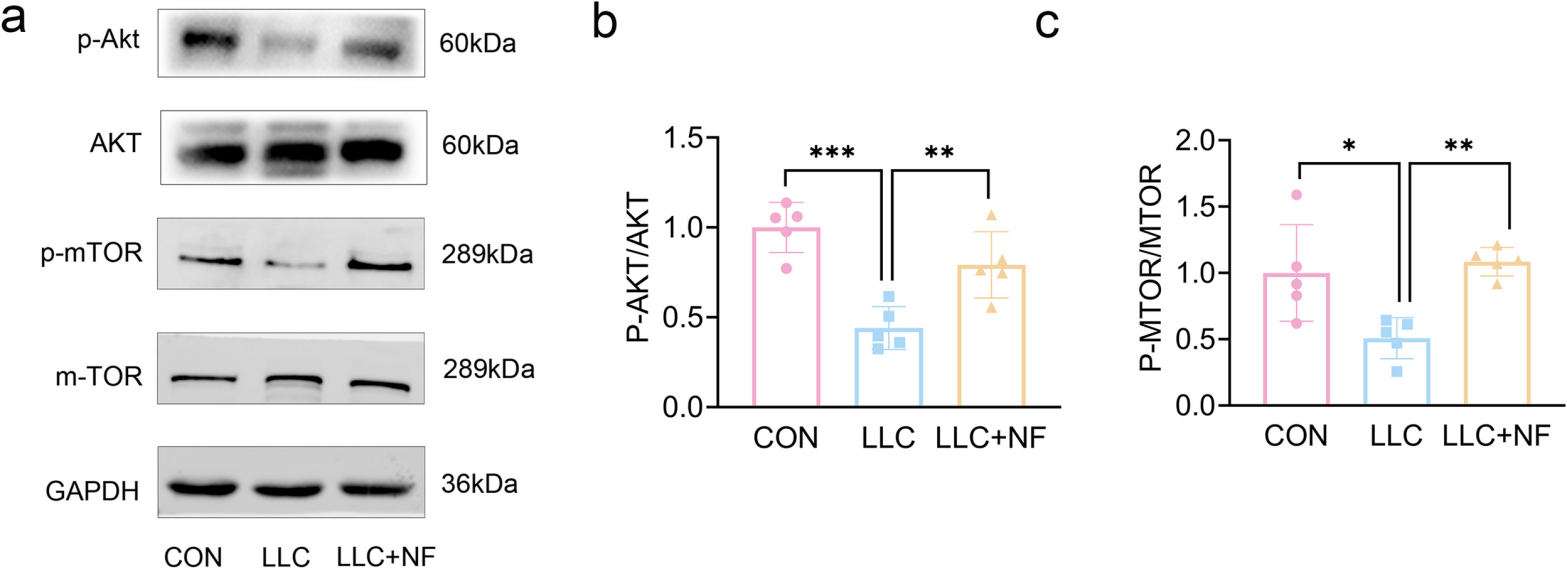
NF alleviated cancer cachexia‐induced muscle atrophy via AKT–mTOR signalling pathway. (a) Representative Western blots showing the expression of p‐AKT, AKT, p‐mTOR and mTOR in the gastrocnemius muscle. (b, c) Relative protein levels in the experiments shown in A. The data were shown as the mean ± SD, *n* = 5. *p ≤ 0.05, **p ≤ 0.01, ***p ≤ 0.001.

KEGG pathway analysis and GO enrichment revealed that PI3K‐AKT signalling pathway may be one of the main signalling for NF against CC. Molecular docking results also showed have good docking scores for both AKT1 and mTOR. It was reported that Akt and mTOR are muscle synthesis factor and they control protein synthesis [21]. As a result, AKT and mTOR protein expression levels were determined. Fortunately, p‐AKT/AKT and p‐mTOR/mTOR protein levels were markedly increased in the LLC + NF group when compared to the LLC group (Figure 6a–c), suggesting that NF may retard CC‐induced muscle atrophy in mice by activating the AKT–mTOR signalling.

### 3.7 NF Directly Binds to HSP90AA1

**FIGURE 7:**
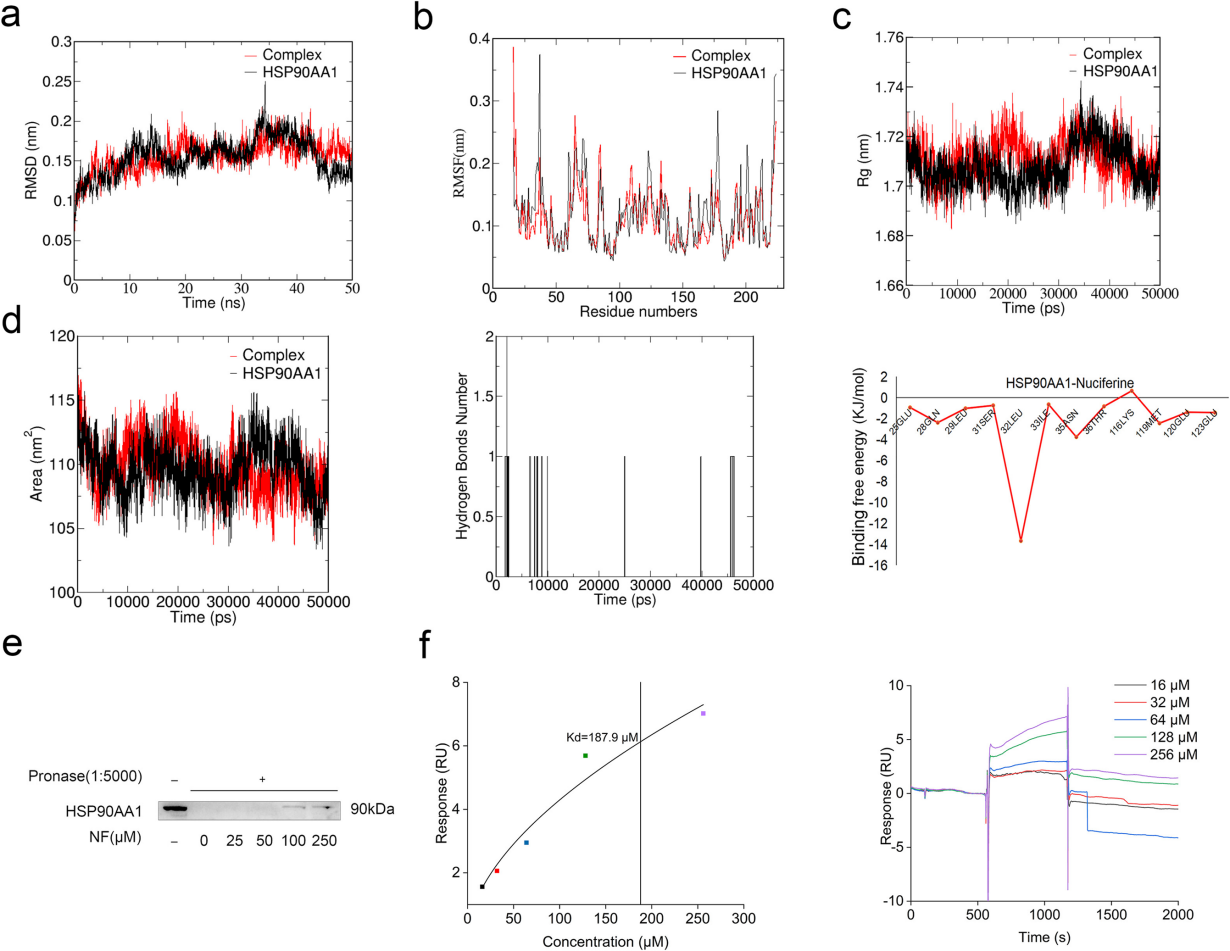
The NF‐HSP90AA1 interaction was verified by molecular dynamics simulation, DARTS and SPR experiments. (a–c) The results of RMSD, RMSF and Rg for complex and HSP90AA1. (d) The SASA, number of hydrogen bonds of the complex and using the last 10‐ns molecular dynamics (MD) simulation locus; the residue energy decomposition of the MM‐PBSA binding energy of the complex was calculated. (e) DARTS assay in C2C12 myotubes. (f) SPR analysis.

According to molecular docking and Western blotting results, we assumed that NF may bind HSP90AA1 and improve CC‐related muscle atrophy. HSP90AA1‐NF complex and HSP90AA1 were selected for MD simulation to examine the binding of NF to HSP90AA1. Protein deviance from its native structure degree has been estimated using the RMSD, which can show how stable the complex is throughout the simulation [22]. For the duration of the simulation, the RMSD of the HSP90AA1‐NF system was less than 0.22 nm throughout the simulation, whereas that of HSP90AA1 was less than 0.26 nm, indicating that the small molecule and protein form a structurally stable complex (Figure 7a).

The RMSF calculates each atom's fluctuation relative to its average position, which can characterize the flexibility and strength of the movement of protein amino acids throughout the simulation. Compared to HSP90AA1, the complex's structure was more stable, and its fluctuations were less pronounced during the whole simulation. Especially, the complex fluctuated in Residues 29–38 range far less than HSP90AA1, indicating that Residues 29–38 may be the key binding residues of NF and HSP90AA1(Figure 7b).

Moreover, we monitored the change in Rg (Radius of rotation), SASA (Solvent Accessible Surface Areas) and hydrogen bind for the HSP90AA1‐NF system (Figure 7c,d). These results further indicate that NF binds stably to HSP90AA1 throughout the simulation process, and hydrogen bonds are not the main force although they are formed transiently during the MD simulation.

We speculated that during the simulation process, factors other than hydrogen bonds may have played a more significant role in maintaining the complex's stability. Next, we computed the cases of binding free energy for the simulated trajectories. The findings demonstrated that the HSP90AA1‐NF complex's binding free energy was −77.281 kJ/mol, which is less than zero (Table S3). Van der Waals force was the primary contributor to the binding of NF to HSP90AA1 among the binding free energy components, which include van der Waals energy, polar solvation energy, electrostatic energy and nonpolar solvation energy. Residue decomposition of the complex's total binding energy was performed (Figure 7d), the main favourable energy contribution of HSP90AA1 to NF binding originated from residues LEU‐32 and ASN‐35, which coincided with the results of the RMSF, suggesting that LEU‐32 and ASN‐35 maybe the key residue sites for the binding of NF to HSP90AA1.

The aforementioned results of MD revealed that NF could bind stably to HSP90AA1 throughout the simulation process and may be the target of NF. However, computational results should be validated with experimental methods because of spurious relationships that may exist from one database to another. Therefore, to further validate the interactions between NF and HSP90AA1, DARTS assay was used to investigate the engagement of NF with HSP90AA1. NF intervention inhibited pronase‐induced HSP90AA1 protein degradation in a concentration‐dependent manner, indicating that NF binds specifically to the HSP90AA1 protein (Figure 7e). Subsequently, SPR affinity assay, another target identification technology, was executed. The sensorgram and fitting curve of NF are displayed in Figure 7f. A strong binding relationship between NF and HSP90AA1 was demonstrated by a Kd of 187.9 μM. These results suggested that NF retarded CC‐induced muscle wasting via directly targeting HSP90AA1.

## 4 Discussion

Cachexia directly results in death for 20%–40% of cancer patients [23]. It was commonly acknowledged that the development of tumour cachexia was largely caused by the decline of skeletal muscle mass, and the best way to control cachectic symptoms was to preserve muscle mass [7]. In order to examine the therapeutic effect of NF on tumour cachexia, muscle strength was measured by GST [24] and WGT [25], and muscle mass was recorded in mice bearing tumours. NF not only improved the muscle strength, as evidenced by the enhancement of limb grip strength, hanging time and frailty score, but also reversed the loss of tumour‐free body weight, skeletal muscle mass, including gastrocnemius and quadriceps and muscle cross sectional area in cachexia mice, indicating that NF effectively alleviated cachexia symptoms in mice bearing tumour.

Network pharmacology is an academic discipline that utilizes database retrieval, network construction and data analysis to forecast drug targets and mechanisms [26]. Using network pharmacology, we evaluated the shared targets between NF and CC. The key genes include AKT1, TNF and HSP90AA1. To further explore the binding of NF to key targets, molecular docking validation was performed. In general, the findings of molecular docking were evaluated using the minimal binding free energy [27]. Through molecular docking, we found that NF bound most stably to HSP90AA1. In addition, the analysis of GO‐enriched functions and KEGG pathways revealed a strong association between these targets and the inflammatory response, upregulation of cell proliferation and the PI3K/Akt signalling pathway, indicating that these mechanisms may be the primary pathway in which NF alleviates muscle atrophy induced by CC.

It is commonly known that inflammation plays a major role in the development of CC [28]. It has been shown that proinflammatory cytokines released by immunological or tumour cells during CC, such as interleukin‐6 (IL‐6), tumour necrosis factor‐α (TNF‐α) and interleukin‐1 (IL‐1), cause muscle atrophy by activation of NF‐κB pathways [7]. Furthermore, some researches have shown that NF inhibits the synthesis of proinflammatory cytokines, including TNF‐α, IL‐1β, IL‐8 and IL‐6, which are regulated by NF‐κB pathways [19, 29, 30]. In addition, it was also discovered that the upregulation of the proinflammatory factor TNF‐α was accompanied by an upregulation of HSP90 [23]. Inflammatory response, HSP90AA1, TNF and inflammatory factor signalling were identified as significant contributors to the antitumour cachexia effect of NF by GO and PPI network analyses. Therefore, we detected whether NF could inhibit the activation of inflammation‐related pathways in the skeletal muscle of mice bearing lung tumours against muscle atrophy. Studies have found that activation of NF‐κB pathway promotes the upregulation of MuRF1 and Atrogin1, which are the muscle‐specific E3 ubiquitin ligases inducing protein degradation, thereby promoting muscle atrophy [31, 32]. Luckily, our findings showed that NF could decrease the levels of protein degradation related markers, including Atrogin1 and MuRF1, and inhibit the release of proinflammatory factors IL‐6 and TNF‐α by inhibiting the activation of HSP90AA1/NF‐κB pathway, indicating that NF, at least in part, alleviated CC muscle atrophy through retarding inflammation‐related protein degradation.

It was reported that CC could inhibit muscle protein synthesis through AKT/mTORC1 signalling suppression [33]. AKT regulates cell differentiation and protein synthesis by regulating mTOR [21], and phosphorylation of AKT is inhibited during muscle atrophy, leading to increase of MyHC degradation [34]. Increased MyHC degradation is widely recognized as an important pathological change in muscle atrophy [35]. GO enrichment analysis showed that cell proliferation was the main biological process, and KEGG analyses also showed that AKT/mTOR pathway might be an important pathway. Therefore, we further performed experimental validation. Happily, the findings indicate that NF retards muscle atrophy by activating Akt/mTOR signalling pathway and decreasing MyHC degradation.

HSP90AA1, heat shock protein 90α Family Class A Member 1, is a highly conserved chaperone protein across species, which plays an important role in cell cycle regulation, gene modification, regulation of DNA damage and the onset and progression of various human cancers [36]. It has been reported that tumour‐released HSP90 and HSP70 trigger muscle atrophy through activation of TLR4 [37], and HSP90 inhibitors can effectively alleviate skeletal muscle consumption caused by CC [38], indicating HSP90 may be the potential therapeutic target for attenuating CC. Among the hub genes predicted by network pharmacology study, the molecular docking analysis revealed that NF had the strongest binding affinity towards HSP90AA1. To further evaluate the stability of NF binding to HSP90AA, MD was conducted to determine the interaction between NF and HSP90AA1 and the binding of NF to the active sites of HSP90AA1. MD is typically used in drug design and target validation and is crucial for understanding dynamic processes and conformational changes of proteins [39]. The HSP90AA1 and NF have a binding energy of −77.281 kJ/mol, according to the results, and the complex remains stable over the simulation. The residues of LEU‐32 and ASN‐35 may be the key sites for the binding of NF to HSP90AA1. Next, we used experiments to verify the accuracy of the calculated target. DARTS, a comparatively rapid and simple method for identifying putative targets for small molecules, depends on the target protein's defence against proteolysis provided by contact with a small molecule [40]. SPR has been widely used in drug‐target interactions [41]. DARTS and SPR results showed that NF binds HSP90AA1 in a dose‐dependent manner. The above results showed that NF attenuates tumour associated muscle atrophy via targeting HSP90AA1.

The limitations of this research should be acknowledged. One of shortcoming of this study is that only male mice were used for the experiments, and no studies were conducted to investigate the role of NF on CC in female mice. In addition, it is unclear that NF regulates the extracellular or intracellular HSP90AA1 for attenuating the muscle wasting in CC, which should be studied in the future by using inhibitors or genetic ablation of HSP90.

## 5 Conclusion

**FIGURE 8:**
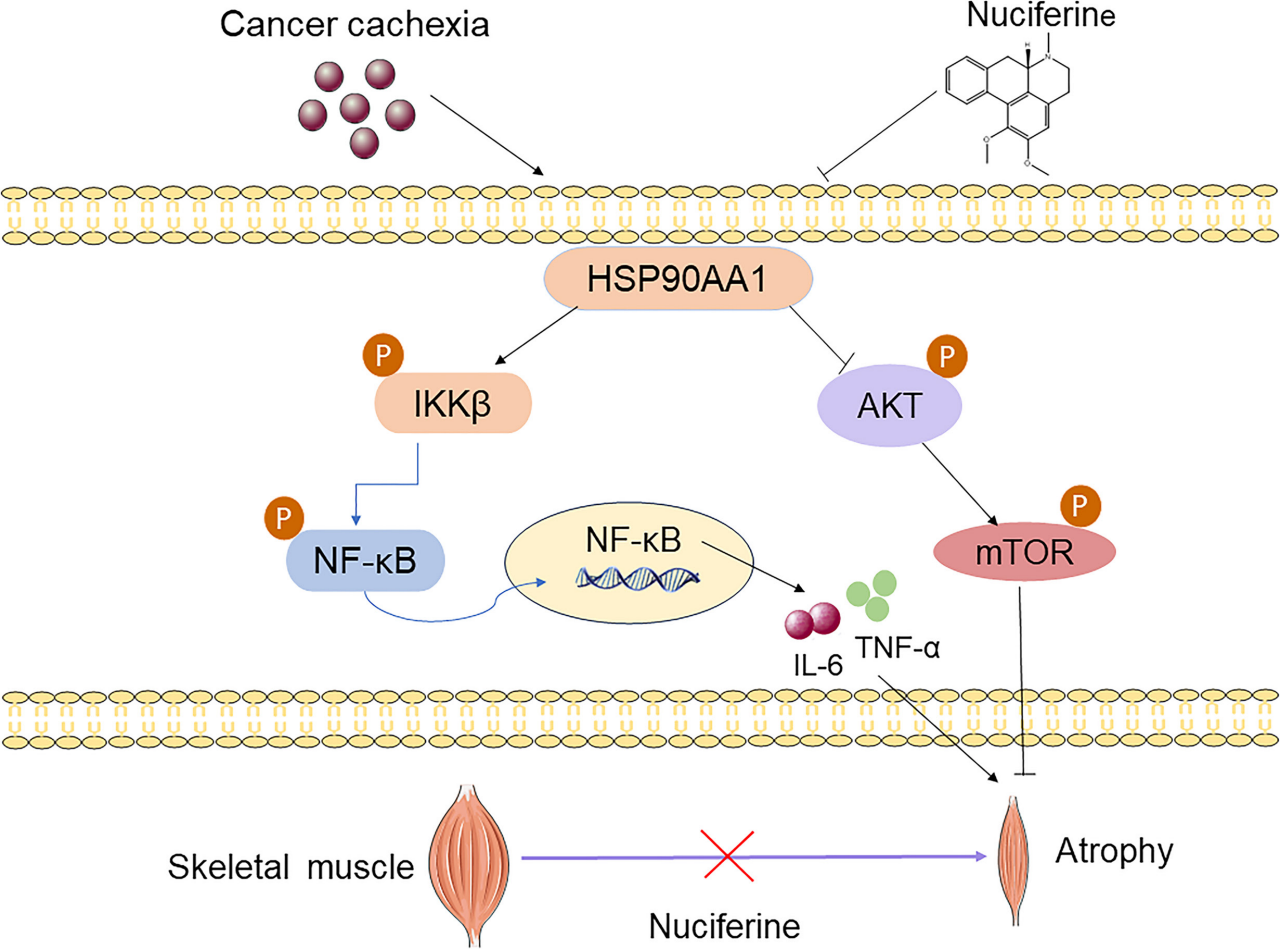
Schematic diagram of the proposed mechanisms underlying the anticachexic effect of NF in mice bearing tumour. NF ameliorates muscle atrophy in mice bearing tumour by regulating NF‐κB and AKT/mTOR signalling pathway via directly binding HSP90AA1.

In conclusion, our study provided novel evidence that NF, an active constituent derived from lotus leaf utilized for its health‐promoting properties in both culinary and herbal medicine, could ameliorate muscle function impairment and skeletal muscle atrophy in CC, as illustrated in Figure 8. According to our experiments, by directly targeting HSP90AA1, NF ameliorates CC‐induced muscle atrophy and function not only by regulating the release of proinflammatory cytokines, including IL‐6 and TNF‐α, via inhibiting NF‐κB pathway, but also by promoting the activation of AKT–mTOR pathway. Thus, our study suggests that NF may be a promising lead compound for the development of new drugs to treat CC.

## Conflicts of Interest

The authors declare no conflicts of interest.

## Supporting information


**Figure S1** NF impacted on the body weight, food intake and tumour volume in mice bearing lung tumour. (a) The body weight. (b) Accumulative food intake. (c) Tumour volume. **(d) Tumour weight.** The data are shown as the **mean ± SD,** *n* = 7 mice/group. ** *p* < 0.01, Control group vs LLC model group.
**Figure S2** Visualization was performed based on molecular docking results. NF docked with (a) PTGS2, (b) MMP2, (c) BCL2, (d) TNF‐α, (e) mTOR, (f) ERBB2.
**Table S1 The top** hub genes with higher degree of connectivity.
**Table S2** Core gene mole Score values.
**Table S3** The averaged binding free energies of the simulated protein‐ligand complex.

## Data Availability

This publication contains all of the data generated or analysed during the study, and upon reasonable request, the corresponding author will make the data available.
