# Supplementary material for: Nuciferine Attenuates Cancer Cachexia‐Induced Muscle Wasting in Mice via HSP90AA1
Source: J Cachexia Sarcopenia Muscle. 2025 Apr 1;16(2):e13777. doi: 10.1002/jcsm.13777 (PMC11961380; doi:10.1002/jcsm.13777)
Supplement: Supplementary file 1 — Figure S1 NF impacted on the body weight, food intake and tumour volume in mice bearing lung tumour. (a) The body weight. (b) Accumulative food intake. (c) Tumour volume. (d) Tumour weight. The data are shown as the mean ± SD, n = 7 mice/group. ** p < 0.01, Control group vs LLC model group. Figure S2 Visualization was performed based on molecular docking results. NF docked with (a) PTGS2, (b) MMP2, (c) BCL2, (d) TNF‐α, (e) mTOR, (f) ERBB2. Table S1 The top hub genes with higher degree of connectivity. Table S2 Core gene mole Score values. Table S3 The averaged binding free energies of the simulated protein‐ligand complex. [file JCSM-16-e13777-s001.docx]

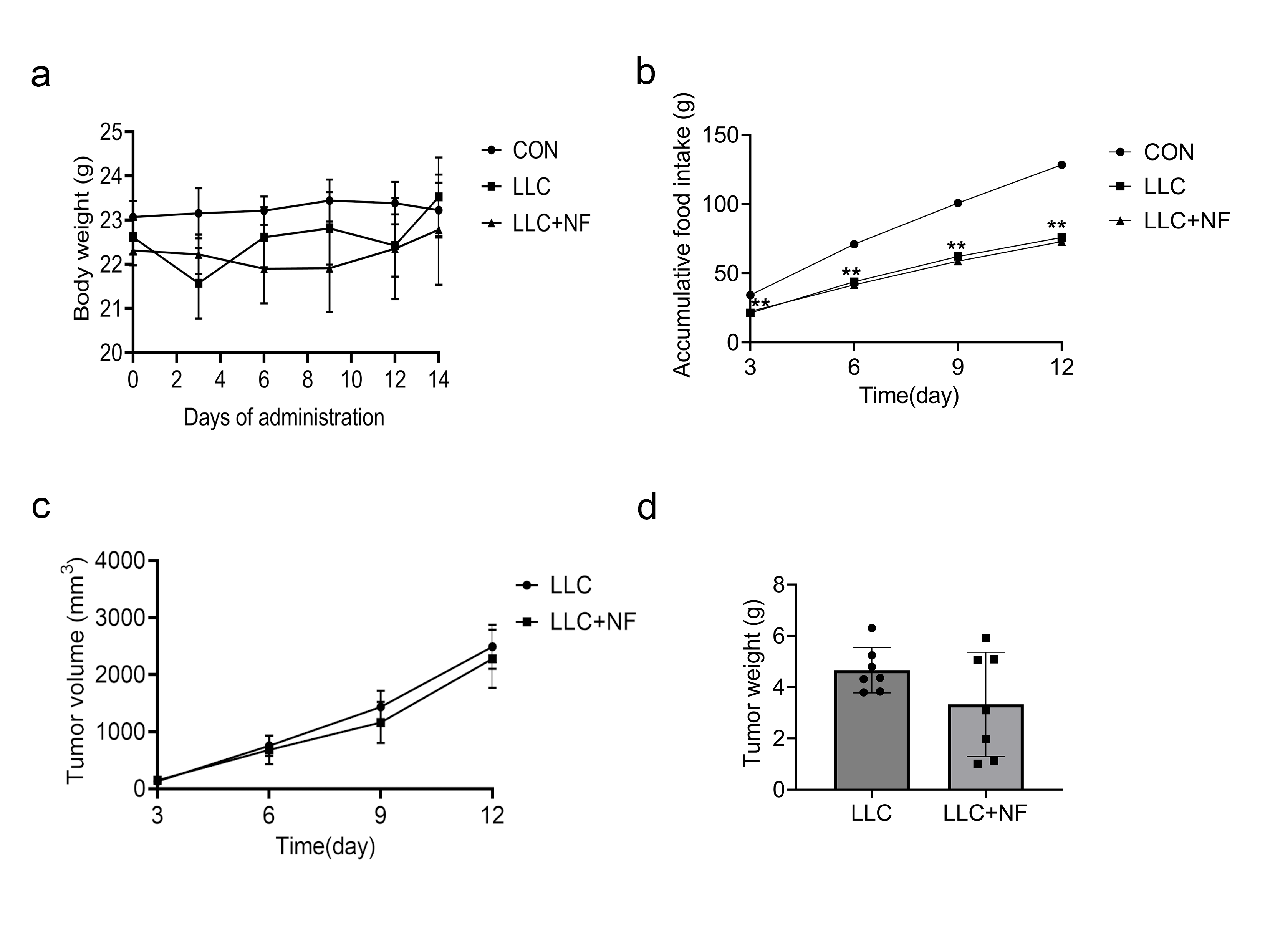


Supplementary Fig1 NF impacted on the body weight, food intake and tumor volume in mice bearing lung tumor. (a) The body weight. (b) Accumulative food intake. (c) Tumor volume. **(d) Tumor weight.** The data are shown as the **mean ± SD,** n =7 mice /group.  **** *P* < 0.01, Control group vs LLC model group.**


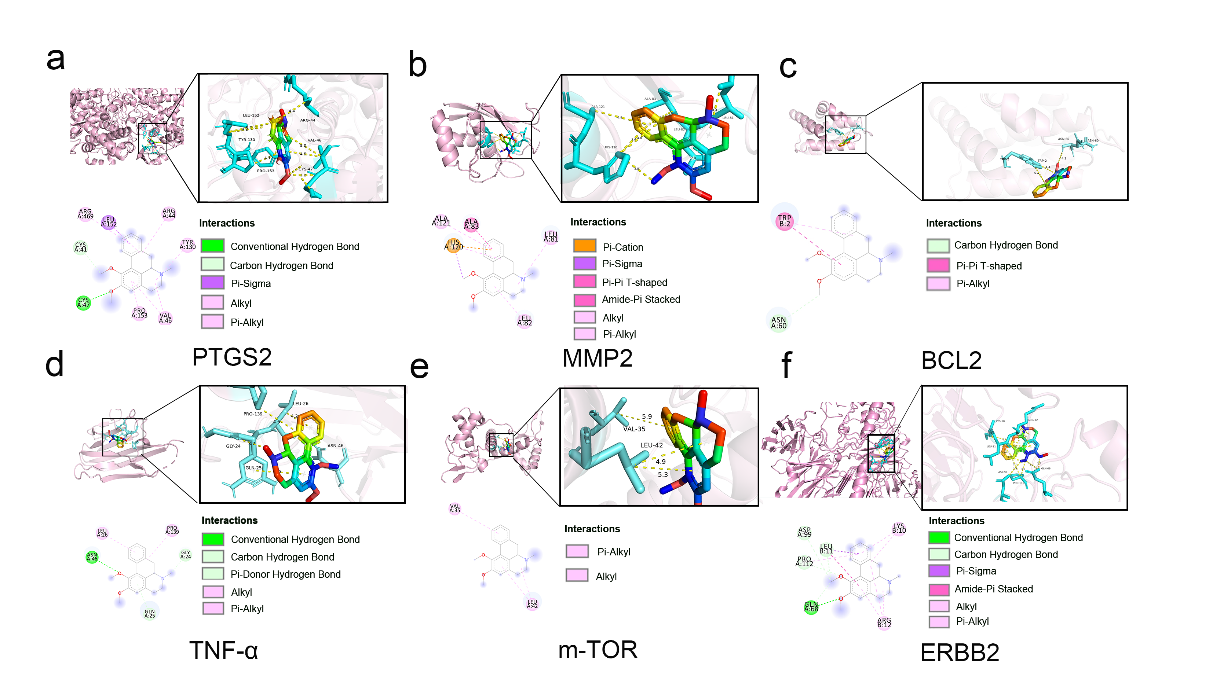


**Supplementary Fig2 Visualization was performed based on molecular docking results. NF docked with (a) PTGS2, (b) MMP2, (c) BCL2, (d) TNF-α, (e) mTOR, (f) ERBB2**

Supplementary Table 1 **The top** hub genes with higher degree of connectivity

| Gene ID | Gene Name | Gene Symbol | Degree |
| --- | --- | --- | --- |
| 207 | AKT serine/threonine | AKT1 | 101 |
| 7124 | Tumor necrosis factor | TNF | 99 |
| 596 | B-Cell Lymphoma 2 | BCL2 | 83 |
| 1956 | epidermal growth factor receptor | EGFR | 82 |
| 4318 | Matrix metallopeptidase 9 | MMP9 | 78 |
| 5743 | prostaglandin G/H synthase 2 | PTGS2 | 72 |
| 3320 | heat shock protein 90 alpha family class A member 1 | HSP90AA1 | 69 |
| 5468 | Peroxisome proliferator-activated receptor gamma | PPARG | 69 |
| 4313 | Matrix metallopeptidase 2 | MMP2 | 62 |
| 2064 | Erb-b2 receptor tyrosine kinase 2 | ERBB2 | 60 |
| 3383 | Intercellular adhesion molecule 1 | ICAM1 | 59 |
| 2475 | mechanistic target of rapamycin kinase | MTOR | 58 |

Supplementary Table2 Core gene mole Score values

| Gene | PDB | Score |
| --- | --- | --- |
| HSP90AA1 | 5h22 | -9.5 |
| EGFR | 3W2S | -8.3 |
| AKT1 | 3os5 | -8.1 |
| PPARG | 8b8w | -7.9 |
| MMP9 | 6ESM | -7.4 |
| ICAM1 | 6S8U | -7.3 |
| PTGS2 | 5f19 | -7.3 |
| MMP2 | 3ayu | -7.2 |
| BCL2 | 5UUK | -7.1 |
| TNFα | 5uuiα | -6.5 |
| mTOR | 7dkl | -6.3 |
| ERBB2 | 3h3b | -6.3 |

Supplementary Table3 The averaged binding free energies of the simulated protein-ligand complex

| Energy type | Energy value (kJ/mol) |
| --- | --- |
| Van der Waal | -92.892 |
| Electrostatic | -9.913 |
| Polar solvation | 39.883 |
| Nonpolar solvation | -14.359 |
| Binding energy | -77.281 |
